# Beyond Our Borders? Public Resistance to Global Genomic Data Sharing

**DOI:** 10.1371/journal.pbio.2000206

**Published:** 2016-11-02

**Authors:** Mary A. Majumder, Robert Cook-Deegan, Amy L. McGuire

**Affiliations:** 1 Center for Medical Ethics and Health Policy, Baylor College of Medicine, Houston, Texas, United States of America; 2 School for the Future of Innovation in Society, Arizona State University Washington Center, Washington, D.C., United States of America; 3 FasterCures, a Center of the Milken Institute, Washington, D.C., United States of America

## Abstract

Prospects have never seemed better for a truly global approach to science to improve human health, with leaders of national initiatives laying out their vision of a worldwide network of related projects. An extensive literature addresses obstacles to global genomic data sharing, yet a series of public polls suggests that the scientific community may be overlooking a significant barrier: potential public resistance to data sharing across national borders. In several large United States surveys, university researchers in other countries were deemed the least acceptable group of data users, and a just-completed US survey found a marked increase in privacy and security concerns related to data access by non-US researchers. Furthermore, diminished support for sharing beyond national borders is not unique to the US, although the limited data from outside the US suggest variation across countries as well as demographic groups. Possible sources of resistance include apprehension about privacy and security protections. Strategies for building public support include making the affirmative case for global data sharing, addressing privacy, security, and other legitimate concerns, and investigating public concerns in greater depth.

## Introduction

Prospects have never seemed better for a truly global approach to science to improve human health. In laying out their vision for President Obama’s Precision Medicine Initiative, Francis Collins and Harold Varmus noted, “efforts should ideally extend beyond our borders, through collaborations with related projects around the world” [1]. This vision is undergirded by a track record of success with a series of projects initially conceived of and carried out as international collaborations, including the Human Genome Project, the International HapMap, ENCODE, and 1000 Genomes.

International collaborations have catalyzed efforts to support global data sharing, beginning with the Bermuda Principles in 1996 [2]. The Toronto International Data Release Agreement built on Bermuda, and these efforts created a solid foundation for more recent expansion through the Global Alliance for Genomics and Health [3–4]. An extensive literature addresses obstacles to global data sharing, especially in the public health domain [5–7], but the scientific community may be overlooking a significant barrier: people’s attitudes about acceptable uses of their data. Public reticence to share data across national borders could derail worldwide scientific and clinical collaborations. We explore the normative foundations of global data sharing and suggest strategies to address the disconnect between scientific and clinical aspirations and the apparent public concern about international data sharing.

## The Case for Global Genomic Data Sharing

The simplest and most compelling argument for global genomic data sharing is instrumental—global sharing enables the best science and ultimately the greatest contributions to human well-being. Collins and Varmus point to data sharing as a way of enlisting “the world’s brightest scientific and clinical minds” in making sense of the anticipated wealth of data [1]. Studies validate the belief that broad data sharing fuels scientific productivity. The human genes initially sequenced and kept as a proprietary resource by Celera Corporation, for example, were cited by 20%–30% fewer research papers—and led to fewer diagnostic tests for those genes—than the genes first mapped by the Human Genome Project and rapidly made public under the Bermuda Principles [8]. Global collaboration is particularly valuable for complex studies of gene—gene and gene—environment interactions [9]. Furthermore, some biomedical research (e.g., rare disease research) is simply not feasible unless case data are collected and shared internationally. Collecting all the cases possible across the globe may be the only way to accumulate enough data to understand a rare disorder. And even for common disorders, international data sharing is important. Genomic variants associated with breast and ovarian cancer in the BRCA1 and BRCA2 genes, for example, are still being discovered more than 20 years after the genes were first sequenced; many millions of people have been tested, and the variants most commonly associated with cancer differ among populations across the globe [10]. Clinical misinterpretation can follow when whole populations are underrepresented in databases, as shown by Manrai et al., for inherited cardiomyopathies [11]. As molecular classification enables ever more refined taxonomies of cancer and other diseases, the case is strengthened for thinking that the best science is global science. Of course, it is important to guard against a simplistic assumption that “more is better”; the best global science depends on the availability of resources for curation and other measures to assure both quality and equity [12].

The idea that the collective human genome is a common heritage of all humanity also resonates globally. Article 1 of the *Universal Declaration on the Human Genome and Human Rights* states: "The human genome underlies the fundamental unity of all members of the human family, as well as the recognition of their inherent dignity and diversity. In a symbolic sense, it is the heritage of humanity" [13]. This universal human rights perspective informs the work of the Global Alliance, including its *Framework for Responsible Sharing of Genomic and Health-Related Data* [4].

Finally, reciprocity is a norm that powerfully influences human behavior [14]. Reciprocity requires that, to the extent data resources in some countries are being made available to qualified researchers globally, other countries have an obligation to reciprocate with openness. DNA sequence databases benefit researchers worldwide. Restrictions that favor local advantage threaten a global regime of scientific sharing. The UK Biobank has made a point of encouraging and providing access to data and samples to qualified researchers across sectors “both in the UK and internationally, without preferential or exclusive access for any user” [15].

## Public Resistance to Global Genomic Data Sharing

A reduction in public support for data sharing when the data will be used to support the profits of private firms has received attention [16–17], and trust in academic researchers drops if they have commercial affiliations [18]. The reluctance to share data across borders has received less attention. Questions about domestic versus international data sharing are rarely included in public opinion surveys. However, US surveys that have addressed this topic have consistently found resistance to sharing across borders. There is no similarly robust data from other countries. The few studies that touch on international data sharing suggest that similar concerns exist outside the US but also that some populations are supportive of international data sharing.

Recent data come from a 2015 survey of 2,601 US adults who were asked, “Would you allow the following types of researchers to use your samples and information for research?” The vast majority favored sharing with researchers at the National Institutes of Health (79%) and university researchers in the US (71%). The level of support for sharing with university researchers in other countries, however, was only 39%, below the 52% level of support for sharing with pharmaceutical or drug company researchers [19]. A 2008 survey of US military veterans found a similar drop in support for sharing data with academic researchers in other countries (43%) compared to US researchers (80%) [20]. Indeed, university researchers in other countries were the least acceptable category of data users in both surveys.

**Fig 1:**
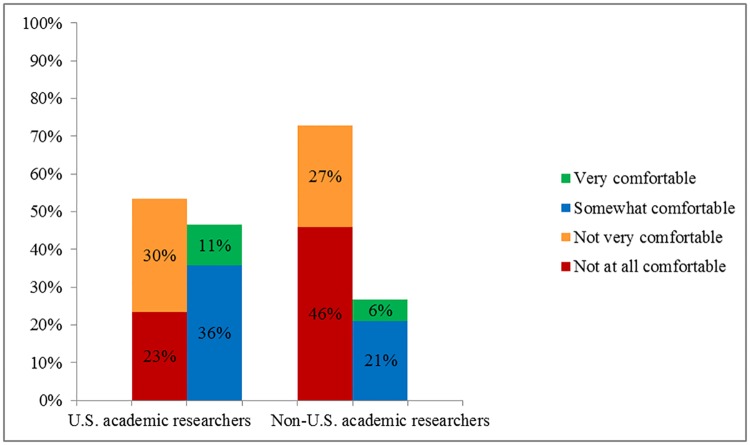
Comfort with health information being accessed by US versus non-US academic researchers (*n* = 1,319).

**Fig 2:**
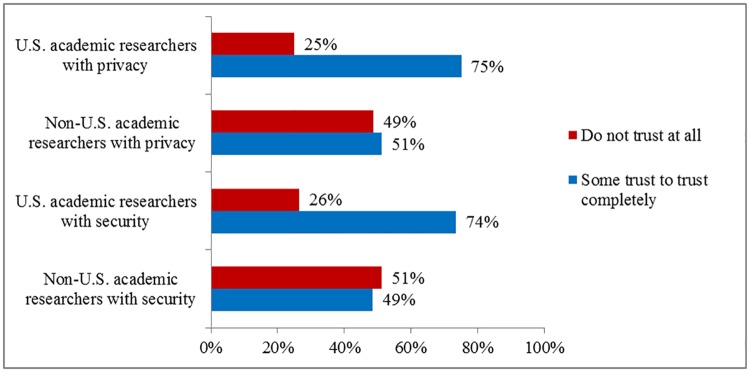
Trust in US versus non-US academic researchers to keep health information private and secure (*n* = 1,319).

In March 2016, we conducted an online survey of 1,319 US adults focused on privacy and security issues using Mechanical Turk, a marketplace for Web-based surveys run by Amazon [21]. We defined “privacy” as “a condition where others have limited access to information about you.” “Security” was defined as “the protections that are in place to keep your information from being seen by people who do not have permission.” Of the respondents, 73% were not at all to not very comfortable with their health information being accessed by academic researchers outside the US, compared to 53% for academic researchers in the US (Fig 1). Moreover, 49% did not trust academic researchers outside the US to keep their health information private (compared to 25% for academic researchers within the US), and 51% did not trust academic researchers outside the US to keep their health information secure (compared to 26% for US researchers) (Fig 2).

Data from outside the US indicate that concern about sharing across borders is not unique to the US, although attitudes vary across countries and demographic groups. A 2007 population survey of 2,400 Finns elicited data on willingness to allow use of research samples depending on the location of companies rather than academic researchers. In that survey, 38% of respondents reported that they would allow use by international companies, while 61% of respondents reported that they would allow use by a Finnish company [22]. However, a 2011 population survey of 3,196 Jordanians found that 23% reported that their decision to donate biological sample(s) and information for biobanking would be positively influenced by “participation of international researchers” (the fifth-ranking positive influence), while 14.8% said their decision would be negatively influenced by this factor (the fourth-ranking negative influence) (the other 59.6% selected “no effect”) [23]. A negative view of sharing with international researchers was associated with increasing age and decreasing educational attainment. Finally, a 2013–2014 Canadian survey used self-identification as a past or potential future donor of tissue samples or genetic data to a biobank or genetic database as an inclusion criterion. Of the 114 individuals completing the survey, 54% selected “international scientific community” when asked to indicate their preferred scope of data sharing (the other options were “a single Canadian institution,” selected by 22.1%, and “undecided,” selected by 23.9%) [24].

## Basis for Privacy and Security Concerns

Privacy and security are frequently mentioned as major sources of public concern related to biobanking and data sharing more generally, and our findings support this emphasis. In the traditional paradigm, anonymization or de-identification is the key to allowing genomic and other health-related data to circulate freely without triggering privacy and security concerns. Recent work suggesting that individuals whose data are included in a genomic database can be identified despite adherence to recognized standards for de-identification challenges this paradigm [25]. Also, anonymization is problematic if privacy is understood to include a right to control access to information about oneself, in addition to an interest in being shielded from risks of harm associated with the disclosure of personal information. Furthermore, even in purely consequentialist terms, the traditional paradigm has weaknesses. Data that are de-identified lose much of their value for research. Many uses of genomic data require continued ability to link to other data about an individual; the individual does not need to be identified in the usual sense for most research uses, but links to clinical records, demographic data, environmental exposures, and health outcomes at an individual level are often needed to draw inferences about genomic variants. The ability to connect data about genomic variants to other outcomes is a touchstone of the Global Alliance [4].

An emerging paradigm accepts a broad conception of privacy, which includes rights to information about uses of data and several forms of control in line with fair information practices, and acknowledges the risk of re-identification. In place of an absolute guarantee against harm, or a claim that re-identification is impossible, it rests on securing broad consent to data sharing (or using a platform that allows for dynamic and granular consent) and continuing efforts to minimize risk (for example, where linkages across records or datasets are critically important, using non-identifying alphanumeric codes to approximate the privacy protection associated with anonymization). Other features include new forms of governance that facilitate ongoing participant engagement, transparency as a means of building trust and as a mark of respect (including transparency about international data sharing), and accountability mechanisms (including sanctions against those who fail to take appropriate steps to secure data or who use data in ways that are not authorized) [4,26–28].

In addition, privacy and security concerns as well as regulatory restrictions on cross-border transfers and data creators’ interests in retaining control have been the impetus for the development of bioinformatics tools that facilitate querying of individual-level data across research sites without centralized storage of those data [29–31]. One of these tools, DataSHIELD, is constructed so that only study-level statistics leave research sites. External researchers are therefore unable to generate results for individual participants.

## Other Possible Sources of Public Resistance

Beyond concerns about privacy and data security, there is a paucity of evidence regarding possible sources of resistance to sharing data with researchers in other countries. Nationalism and concerns about economic competitiveness may be additional factors. Investment in biomedical research is often promoted as an engine of national economic growth and competitive advantage [32]. The link between prohibitions on cross-border data sharing and the promotion of national interests in biotechnology prowess is not direct, however, and cooperation as well competition may advance economic development.

General distrust based on concerns about use in controversial research or potential exploitation may also be sources of resistance. Even pure data research may be highly controversial if, for example, it involves linking a stigmatized condition to a particular population or social group. Residents of low- and middle-income countries and indigenous peoples have distinctive concerns about exploitation. Exploitation encompasses instances of “helicopter genetics,” the descent of scientists from developed countries on developing countries to carry out research that violates standards of research ethics, as well as the use of data without proper credit to local data collectors and a lack of benefit sharing with local populations that contribute data [5–7].

## Strategies to Promote Global Genomic Data Sharing

The aspirations for global genomic data sharing are laudable and important. They may nonetheless confront public reluctance to share data across borders. Building public support requires both improved communication about benefits and attention to privacy, security, and other legitimate concerns. First, the benefits of global data sharing are not immediately obvious—the affirmative case for global sharing of genomic and other data must be made. The common heritage idea may resonate in regions of the world where solidarity is an important cultural norm but is unlikely to prove as successful in countries like the US, where individualism and patriotism are core values. The notion that the best science is global science should have more universal appeal. Those focused on rare disease and precision medicine research have a compelling case that progress can only be made if data are pooled globally. A strong, direct, reciprocity-based argument is available where instances of in-country researchers benefiting from another country’s resource can be cited, and it may be possible to activate reciprocity in a more general way by conveying that “free riders” threaten the demise of nascent pro-sharing norms that benefit all.

A second set of strategies can address potential sources of resistance. In countries such as the US, built through immigration, it may be helpful for leaders of public health and research funding agencies, researchers, and patient groups to communicate that each “nation of nations” [33] has a stake in efforts to capture data from many populations worldwide. Interpreting the BRCA variant of a woman born in Iceland who moved to New York, or the gene variant found in a child with epilepsy from Malta or Malawi, may depend on such pooling of data. More broadly, advocates for global data sharing can develop talking points that connect sharing to scientific leadership and fulfillment of national economic aspirations. We note that the legitimacy of this strategy rests on an investment by sponsors of global initiatives to ensure that their projects are structured so that champions in all participating nations have a leadership role and some control, and that benefits are fairly shared. In this respect, the work of the Worldwide Antimalarial Resistance Network appears exemplary [12]. Advocates should also consider briefing political leaders on the desirability of emphasizing cooperation as well as competition in narratives that link biomedical research to national economic growth.

Advocates can address privacy and security concerns by building strong and secure platforms for sharing while also providing information to the public about the stringency of protections that pertain to users in other countries. They must also remedy gaps by pushing for stronger laws and other measures to address vulnerabilities and to penalize unauthorized re-identification and breaches of privacy. Most countries do not strictly prohibit export of biospecimens or data, but many impose restrictions such as compliance with EU standards for receipt of data and biospecimens, de-identification (typically compatible with use of a non-identifying code) or anonymization of data before transfer, and review and approval of the proposed transfer by a research ethics board [9,34]. A few countries require a special permit or collaboration with a local researcher, and requests for access to genetic data may be subject to additional approval requirements (e.g., China, France, India, Mexico, Nigeria) [34].

Current efforts by the Global Alliance and others to develop common review standards, procedures, and conditions for exchanging data should improve efficiency without compromising the role ethics review boards can play in ensuring that privacy, security, and concerns about potentially controversial or exploitative research are addressed and are perceived as being addressed by the public and relevant subpopulations [4,35]. Among other things, review boards can require specification of the terms of use in an agreement that includes sanctions for violations and is enforceable across jurisdictions [9]. Such measures must do more work when transfers are from a region or country that has strong data protections to a country, such as the US, that does not. In the absence of strong general legal protections, advocates can push for targeted laws or standards that protect against the misuse of shared data with stringent penalties for bad actors. We acknowledge that laws and standards are subject to change and that implementation and enforcement efforts may falter. Hence, we strongly support the emerging privacy paradigm, with its emphasis on ongoing engagement, transparency, and accountability [4]. Furthermore, we recognize that some individuals and communities may be justified in constraining cross-border data sharing in light of their analysis of risks and benefits.

Finally, further study is needed to complete the picture by capturing public opinion in more countries and to understand why support diminishes for sharing data and materials across borders in some countries and demographic groups. What are the sources of concern, and what are the most effective responses? The strategies we outline are sensible and have little or no down-side risk, but they are based on very limited evidence. Better understanding of the causes for public concern might lead to the development of more effective, targeted strategies to build public support for an international information commons to advance biomedical research.

## Supporting Information

S1 Table US vs Outside Researchers Frequencies. (PDF)

S1 Text Health Privacy MTurk Survey Methods. (DOCX)

S2 Text Health Privacy MTurk Survey Demographics. (DOCX)

S1 Data Data US vs Outside Researchers Raw Data. (XLSX)
