# Supplementary material for: Beyond Our Borders? Public Resistance to Global Genomic Data Sharing
Source: PLoS Biol. 2016 Nov 2;14(11):e2000206. doi: 10.1371/journal.pbio.2000206 (PMC5091881; doi:10.1371/journal.pbio.2000206)
Supplement: S1 Table — (PDF) [file pbio.2000206.s001.pdf]

```
FREQUENCIES VARIABLES=Comfortable_reseachers Comfortable_outsideresearchers Pu
rpose_Usresearch
    Purpose_Outsideresearch Private_Usresearcher Private_Outresearcher Securit
y_Usresearcher
    Security_Outresearcher
  /STATISTICS=MEAN MEDIAN MODE SUM
  /BARCHART PERCENT
  /ORDER=ANALYSIS.
```

Frequencies

Notes

Output Created		02-MAY-2016 12:47:04
Comments		
Input	Data	C:\Users\hpeoples\Box Sync\McGuire\Rothstein_Privacy\Results & Data Analysis\Analysis\ELSI Survey Complete SPSS Data Set.sav
	Active Dataset	DataSet1
	Filter	<none>
	Weight	<none>
	Split File	<none>
	N of Rows in Working Data File	1319
Missing Value Handling	Definition of Missing	User-defined missing values are treated as missing.
	Cases Used	Statistics are based on all cases with valid data.
Syntax		FREQUENCIES VARIABLES=Comfortable_reseachers Comfortable_outsideresearchers Purpose_Usresearch Purpose_Outsideresearch Private_Usresearcher Private_Outresearcher Security_Usresearcher Security_Outresearcher /STATISTICS=MEAN MEDIAN MODE SUM /BARCHART PERCENT /ORDER=ANALYSIS.
Resources	Processor Time	00:00:00.99
	Elapsed Time	00:00:00.75

Statistics

		How comfortable are you / would you be with your health information being accessed by the following entities? Academic researchers in the United States	How comfortable are you / would you be with your health information being accessed by the following entities? Academic researchers outside of the United States	How comfortable are you / would you be with your health information being accessed for the following purposes? Academic research in the United States	How comfortable are you / would you be with your health information being accessed for the following purposes? Academic research outside of the United States	How much do you / would you trust the following entities to keep your health information private? Academic researchers in the United States	How much do you / would you trust the following entities to keep your health information private? Academic researchers outside of the United States	How much do you / would you trust the following entities to keep your health information secure? Academic researchers in the United States	How much do you / would you trust the following entities to keep your health information secure? Academic researchers outside of the United States
N	Valid	1319	1319	1319	1319	1319	1319	1319	1319
	Missing	0	0	0	0	0	0	0	0
Mean		2.3412	1.8560	2.3730	1.9075	2.2972	1.7998	2.2297	1.7445
Median		2.0000	2.0000	2.0000	2.0000	2.0000	2.0000	2.0000	1.0000
Mode		3.00	1.00	3.00	1.00	2.00	1.00	2.00	1.00
Sum		3088.00	2448.00	3130.00	2516.00	3030.00	2374.00	2941.00	2301.00

Frequency Table

How comfortable are you / would you be with your health information being accessed by the following entities?Academic researchers in the United States

		Frequency	Percent	Valid Percent	Cumulative Percent
Valid	Not at all Comfortable	307	23.3	23.3	23.3
	Not very Comfortable	398	30.2	30.2	53.4
	Somewhat Comfortable	471	35.7	35.7	89.2
	Very Comfortable	143	10.8	10.8	100.0
	Total	1319	100.0	100.0	

How comfortable are you / would you be with your health information being accessed by the following entities? Academic researchers outside of the United States

		Frequency	Percent	Valid Percent	Cumulative Percent
Valid	Not at all Comfortable	615	46.6	46.6	46.6
	Not very Comfortable	353	26.8	26.8	73.4
	Somewhat Comfortable	277	21.0	21.0	94.4
	Very Comfortable	74	5.6	5.6	100.0
	Total	1319	100.0	100.0	

How comfortable are you / would you be with your health information being accessed for the following purposes?Academic research in the United States

		Frequency	Percent	Valid Percent	Cumulative Percent
Valid	Not at all Comfortable	305	23.1	23.1	23.1
	Not very Comfortable	373	28.3	28.3	51.4
	Somewhat Comfortable	485	36.8	36.8	88.2
	Very Comfortable	156	11.8	11.8	100.0
	Total	1319	100.0	100.0	

How comfortable are you / would you be with your health information being accessed for the following purposes?Academic research outside of the United States

		Frequency	Percent	Valid Percent	Cumulative Percent
Valid	Not at all Comfortable	601	45.6	45.6	45.6
	Not very Comfortable	333	25.2	25.2	70.8
	Somewhat Comfortable	291	22.1	22.1	92.9
	Very Comfortable	94	7.1	7.1	100.0
	Total	1319	100.0	100.0	

How much do you / would you trust the following entities to keep your health information private? Academic researchers in the United States

		Frequency	Percent	Valid Percent	Cumulative Percent
Valid	Do Not Trust At All1	328	24.9	24.9	24.9
	2	421	31.9	31.9	56.8
	3	420	31.8	31.8	88.6
	Trust Completely4	150	11.4	11.4	100.0
	Total	1319	100.0	100.0	

How much do you / would you trust the following entities to keep your health information private? Academic researchers outside of the United States

		Frequency	Percent	Valid Percent	Cumulative Percent
Valid	Do Not Trust At All1	642	48.7	48.7	48.7
	2	373	28.3	28.3	77.0
	3	230	17.4	17.4	94.4
	Trust Completely4	74	5.6	5.6	100.0
	Total	1319	100.0	100.0	

How much do you / would you trust the following entities to keep your health information secure? Academic researchers in the United States

		Frequency	Percent	Valid Percent	Cumulative Percent
Valid	Do Not Trust At All1	348	26.4	26.4	26.4
	2	457	34.6	34.6	61.0
	3	377	28.6	28.6	89.6
	Trust Completely4	137	10.4	10.4	100.0
	Total	1319	100.0	100.0	

How much do you / would you trust the following entities to keep your health information secure? Academic researchers outside of the United States

		Frequency	Percent	Valid Percent	Cumulative Percent
Valid	Do Not Trust At All1	677	51.3	51.3	51.3
	2	375	28.4	28.4	79.8
	3	194	14.7	14.7	94.5
	Trust Completely4	73	5.5	5.5	100.0
	Total	1319	100.0	100.0	

Bar Chart

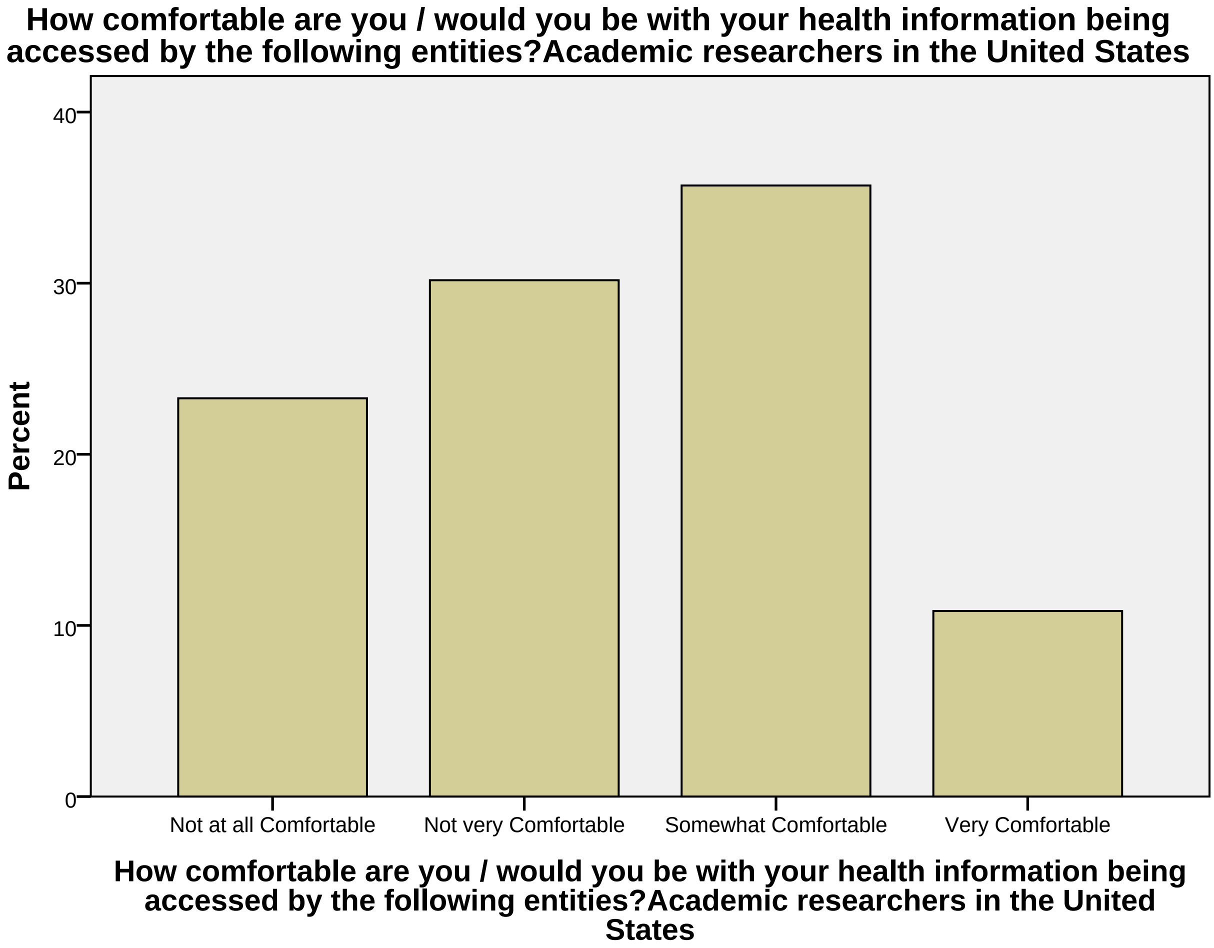
How comfortable are you / would you be with your health information being accessed by the following entities?Academic researchers in the United States
Percent
40
30
20
10
0
Not at all Comfortable
Not very Comfortable
Somewhat Comfortable
Very Comfortable
How comfortable are you / would you be with your health information being accessed by the following entities?Academic researchers in the United States

How comfortable are you / would you be with your health information being accessed by the following entities? Academic researchers outside of the United States

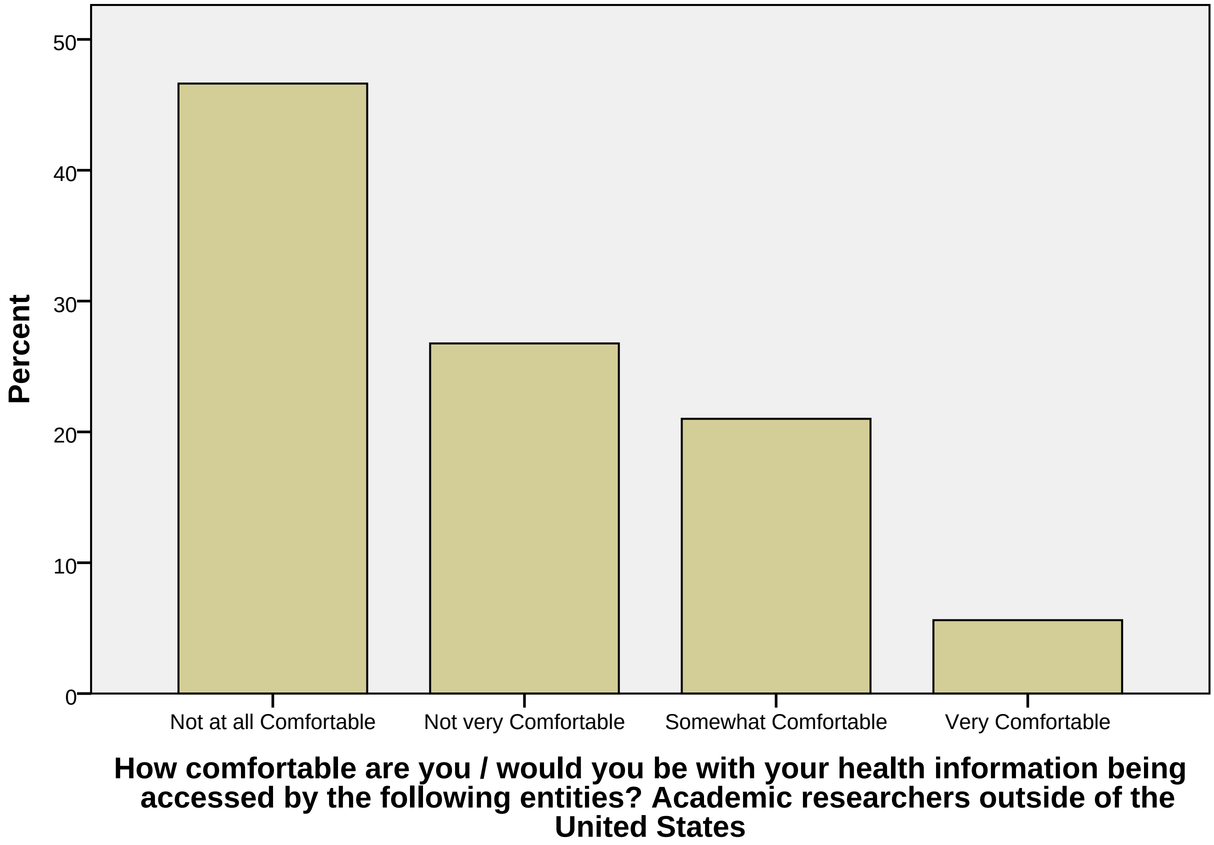

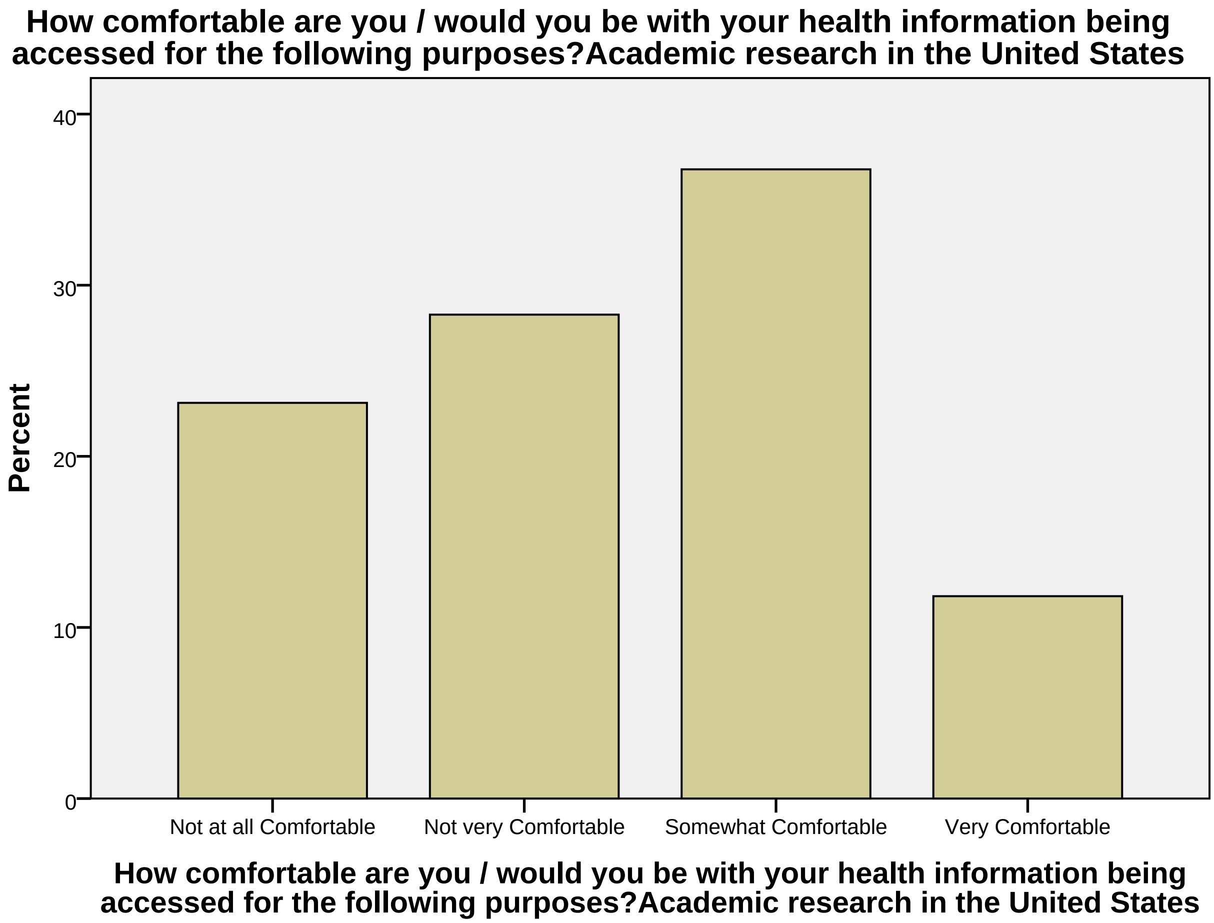
How comfortable are you / would you be with your health information being accessed for the following purposes?Academic research in the United States
Percent
40
30
20
10
0
Not at all Comfortable
Not very Comfortable
Somewhat Comfortable
Very Comfortable
How comfortable are you / would you be with your health information being accessed for the following purposes?Academic research in the United States

How comfortable are you / would you be with your health information being accessed for the following purposes?Academic research outside of the United States

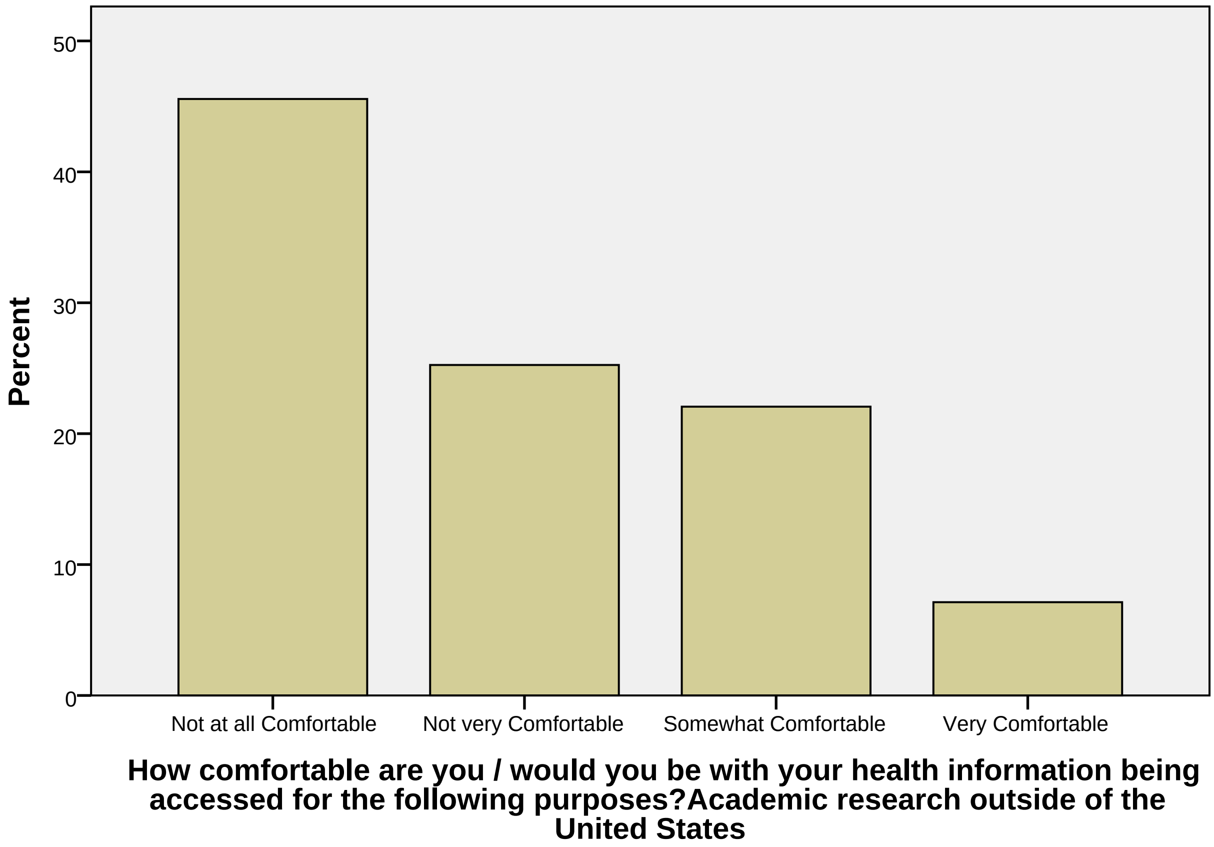

How much do you / would you trust the following entities to keep your health information private? Academic researchers in the United States

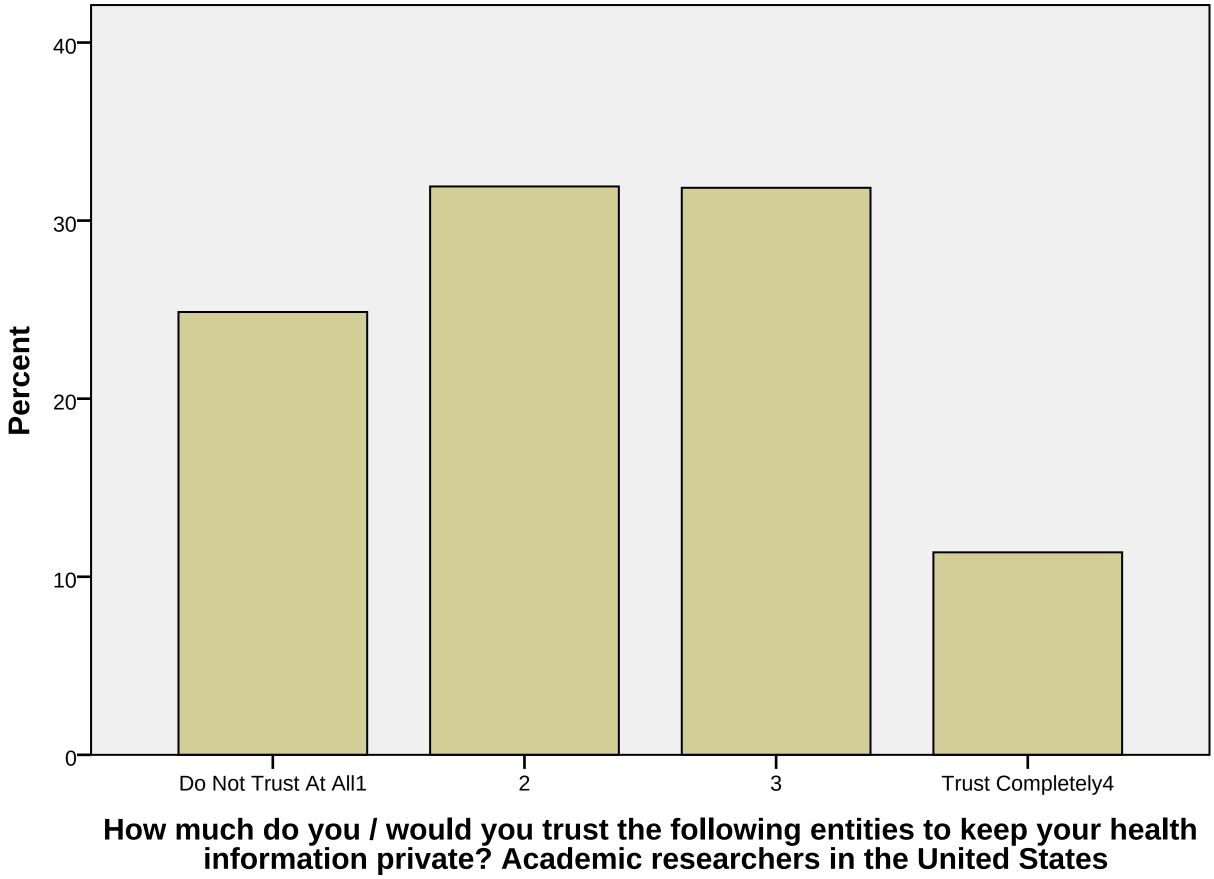

How much do you / would you trust the following entities to keep your health information private? Academic researchers outside of the United States

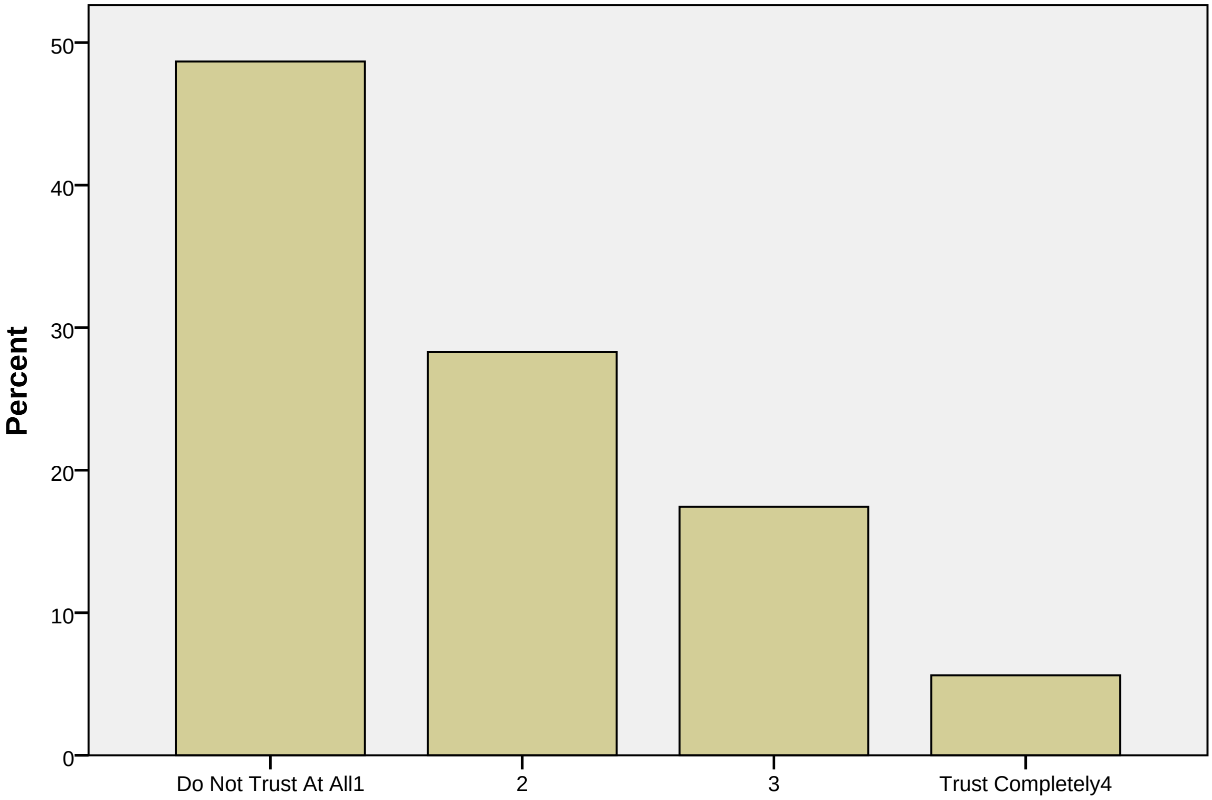

How much do you / would you trust the following entities to keep your health information private? Academic researchers outside of the United States

How much do you / would you trust the following entities to keep your health information secure? Academic researchers in the United States

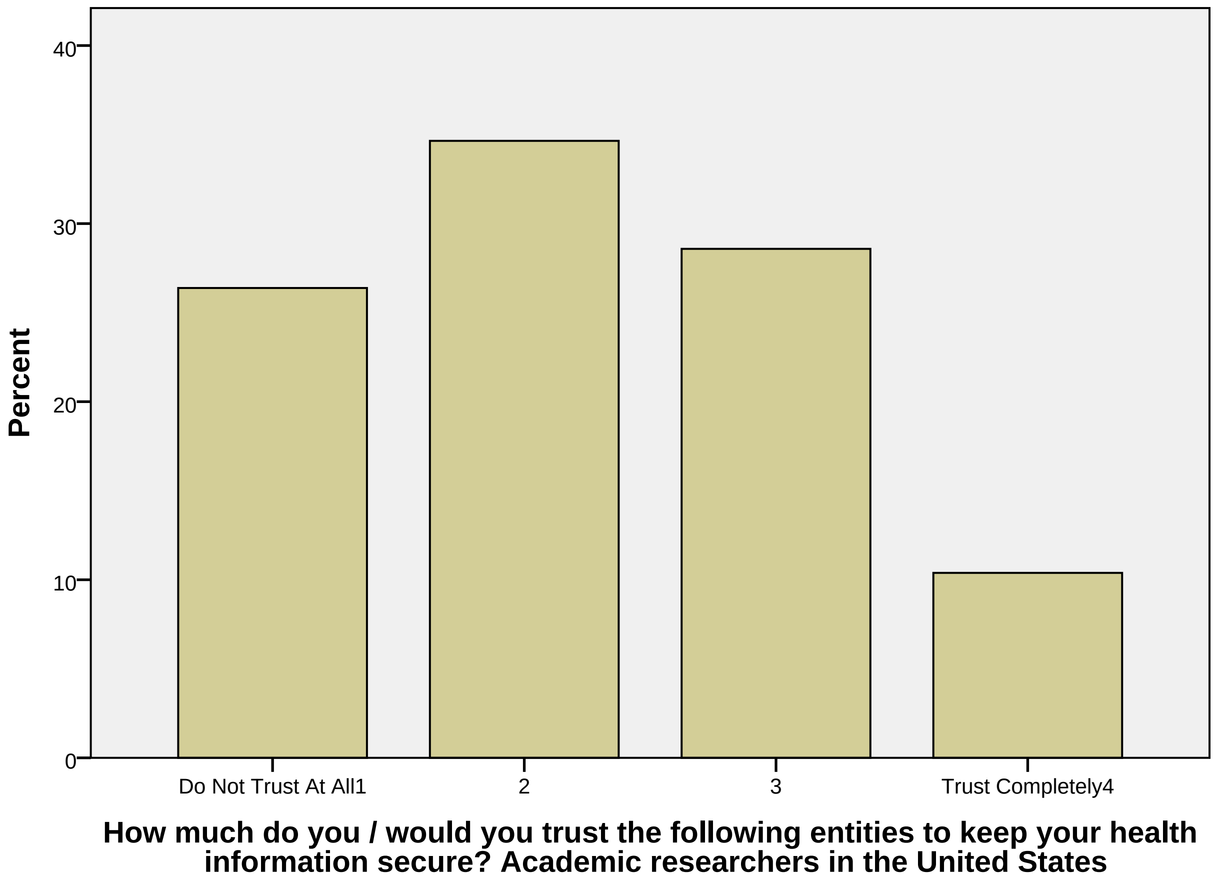

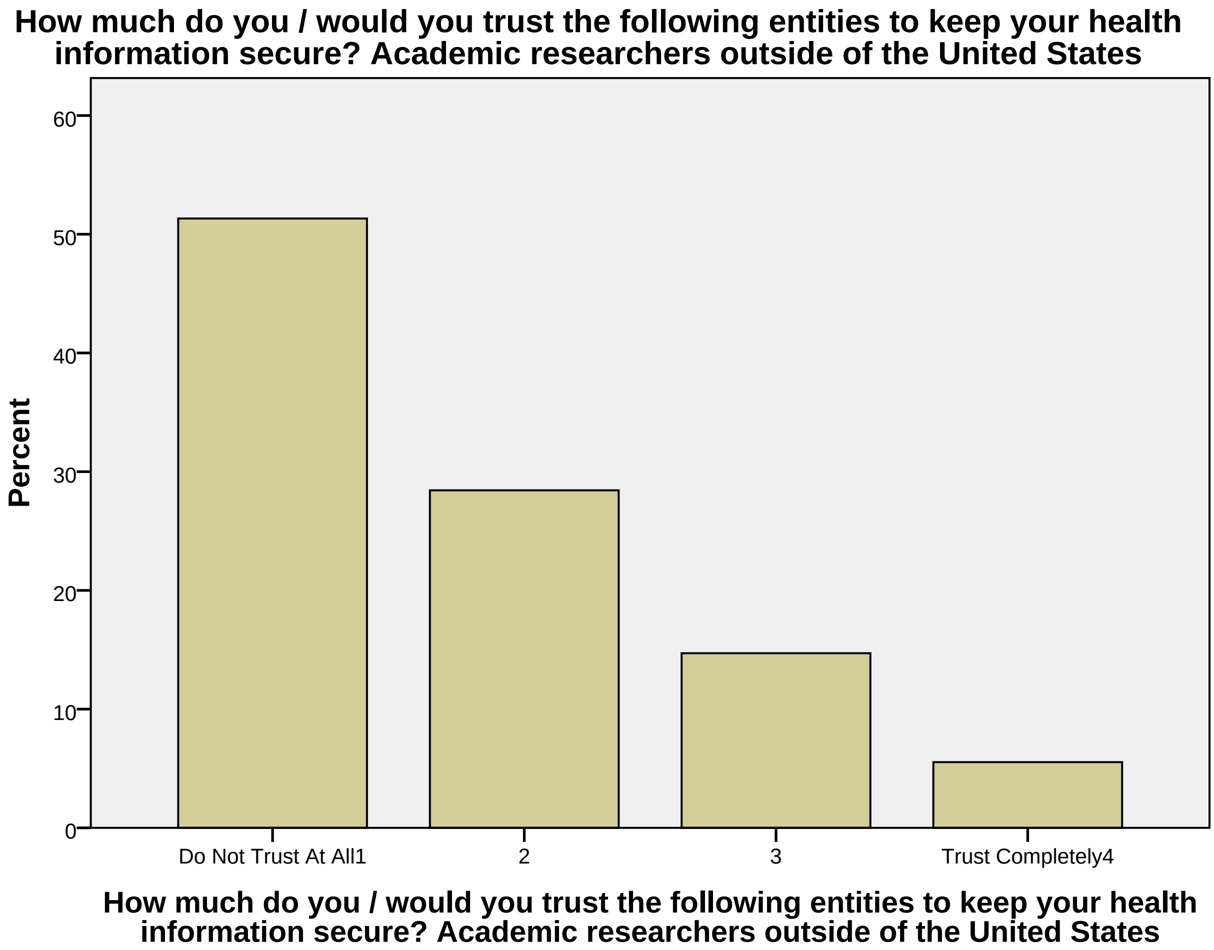


```
CROSSTABS
 /TABLES=Comfortable_reseachersBY Comfortable_outsideresearchers
 /FORMAT=AVALUE TABLES
 /STATISTICS=CHISQ
 /CELLS=COUNT
 /COUNT ROUND CELL
 /BARCHART.
```

Crosstabs

Notes

Output Created		02-MAY-2016 12:52:44
Comments		
Input	Data	C:\Users\hpeoples\Box Sync\McGuire\Rothstein_Privacy\Results & Data Analysis\Analysis\ELSI Survey Complete SPSS Data Set.sav
	Active Dataset	DataSet1
	Filter	<none>
	Weight	<none>
	Split File	<none>
	N of Rows in Working Data File	1319
Missing Value Handling	Definition of Missing	User-defined missing values are treated as missing.
	Cases Used	Statistics for each table are based on all the cases with valid data in the specified range(s) for all variables in each table.
Syntax		CROSSTABS<br>/TABLES=Comfortable_reseachers BY Comfortable_outsideresearchers<br>/FORMAT=AVALUE TABLES<br>/STATISTICS=CHISQ<br>/CELLS=COUNT<br>/COUNT ROUND CELL<br>/BARCHART.
Resources	Processor Time	00:00:00.14
	Elapsed Time	00:00:00.12
	Dimensions Requested	2
	Cells Available	349496

Case Processing Summary

	Cases					
	Valid		Missing		Total	
	N	Percent	N	Percent	N	Percent
How comfortable are you / would you be with your health information being accessed by the following entities?Academic researchers in the United States * How comfortable are you / would you be with your health information being accessed by the following entities? Academic researchers outside of the United States	1319	100.0%	0	0.0%	1319	100.0%

How comfortable are you / would you be with your health information being accessed by the following entities?Academic researchers in the United States * How comfortable are you / would you be with your health information being accessed by the following entities? Academic researchers outside of the United States Crosstabulation

Count

		How comfortable are you / would you be with your health information being accessed by the following entities? Academic researchers outside of the United States				Total
		Not at all Comfortable	Not very Comfortable	Somewhat Comfortable	Very Comfortable	
How comfortable are you / would you be with your health information being accessed by the following entities?Academic researchers in the United States	Not at all Comfortable	305	0	2	0	307
	Not very Comfortable	212	172	13	1	398
	Somewhat Comfortable	86	157	222	6	471
	Very Comfortable	12	24	40	67	143
Total		615	353	277	74	1319

Chi-Square Tests

	Value	df	Asymptotic Significance (2-sided)
Pearson Chi-Square	1211.802[a]	9	.000
Likelihood Ratio	1106.877	9	.000
Linear-by-Linear Association	669.410	1	.000
N of Valid Cases	1319		

a. 0 cells (0.0%) have expected count less than 5. The minimum expected count is 8.02.

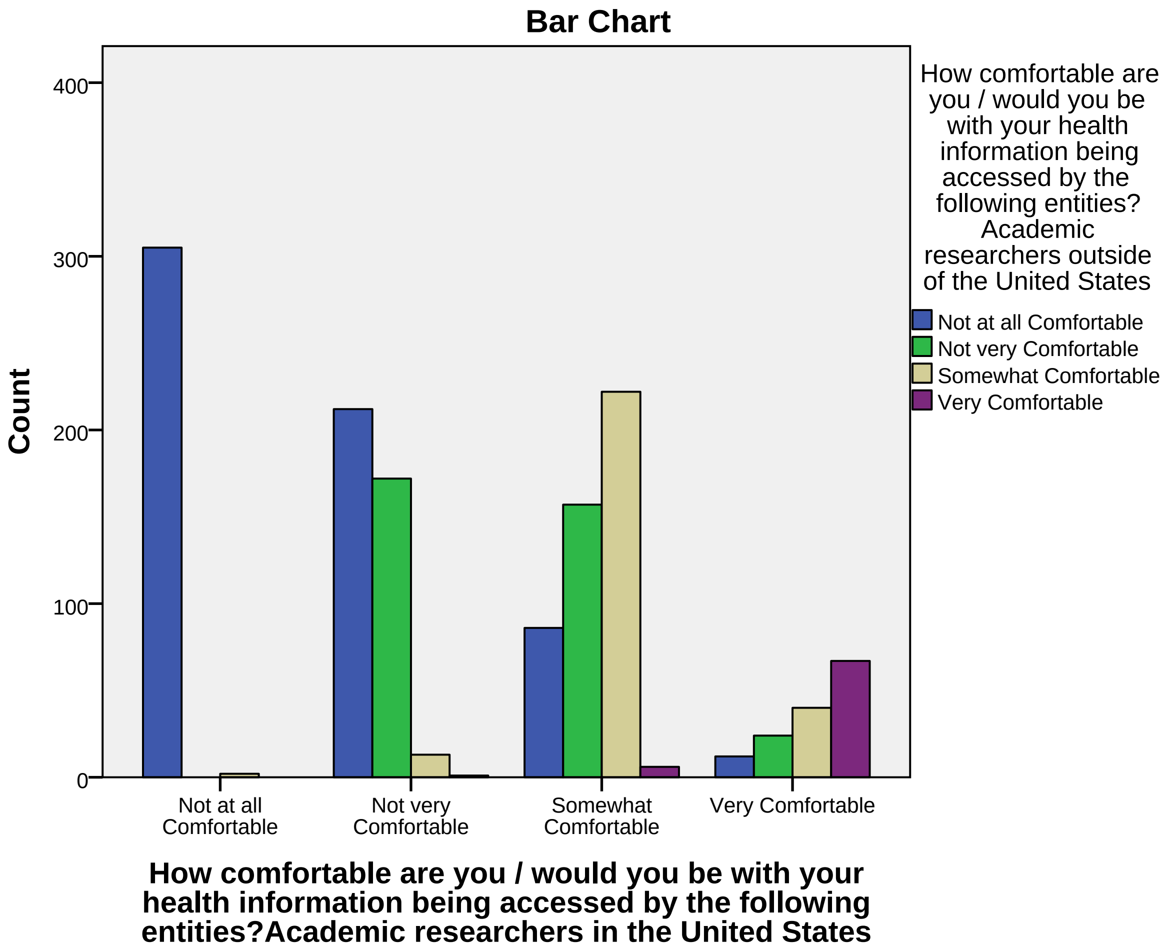


```
CROSSTABS
  /TABLES=Purpose_Outsideresearch BY Purpose_Usresearch
  /FORMAT=AVALUE TABLES
  /STATISTICS=CHISQ
  /CELLS=COUNT
  /COUNT ROUND CELL
  /BARCHART.
```

Crosstabs

Notes

Output Created		02-MAY-2016 12:53:02
Comments		
Input	Data	C:\Users\hpeoples\Box Sync\McGuire\Rothstein_Privacy\Results & Data Analysis\Analysis\ELSI Survey Complete SPSS Data Set.sav
	Active Dataset	DataSet1
	Filter	<none>
	Weight	<none>
	Split File	<none>
	N of Rows in Working Data File	1319
Missing Value Handling	Definition of Missing	User-defined missing values are treated as missing.
	Cases Used	Statistics for each table are based on all the cases with valid data in the specified range(s) for all variables in each table.
Syntax		CROSSTABS<br><br>/TABLES=Purpose_Outsideresearch BY Purpose_Usresearch<br>/FORMAT=AVALUE TABLES<br>/STATISTICS=CHISQ<br>/CELLS=COUNT<br>/COUNT ROUND CELL<br>/BARCHART.
Resources	Processor Time	00:00:00.14
	Elapsed Time	00:00:00.11
	Dimensions Requested	2
	Cells Available	349496

Case Processing Summary

	Cases					
	Valid		Missing		Total	
	N	Percent	N	Percent	N	Percent
How comfortable are you / would you be with your health information being accessed for the following purposes?Academic research outside of the United States * How comfortable are you / would you be with your health information being accessed for the following purposes? Academic research in the United States	1319	100.0%	0	0.0%	1319	100.0%

How comfortable are you / would you be with your health information being accessed for the following purposes?Academic research outside of the United States * How comfortable are you / would you be with your health information being accessed for the following purposes?Academic research in the United States Crosstabulation

Count

		How comfortable are you / would you be with your health information being accessed for the following purposes?Academic research in the United States				Total
		Not at all Comfortable	Not very Comfortable	Somewhat Comfortable	Very Comfortable	
How comfortable are you / would you be with your health information being accessed for the following purposes?Academic research outside of the United States	Not at all Comfortable	296	193	100	12	601
	Not very Comfortable	4	158	153	18	333
	Somewhat Comfortable	2	21	226	42	291
	Very Comfortable	3	1	6	84	94
Total		305	373	485	156	1319

Chi-Square Tests

	Value	df	Asymptotic Significance (2-sided)
Pearson Chi-Square	1212.790[a]	9	.000
Likelihood Ratio	1058.796	9	.000
Linear-by-Linear Association	641.432	1	.000
N of Valid Cases	1319		

a. 0 cells (0.0%) have expected count less than 5. The minimum expected count is 11.12.

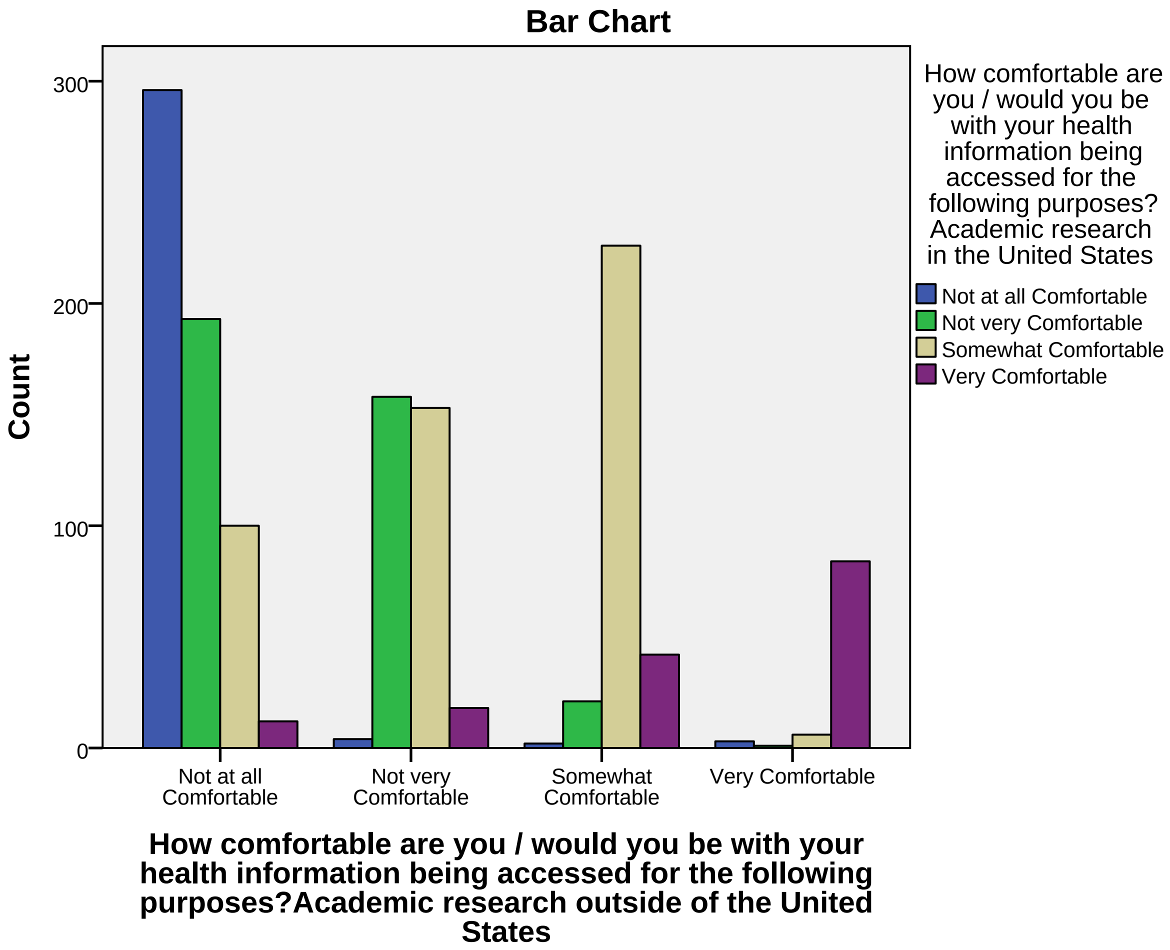


```
CROSSTABS
 /TABLES=Private_OutresearcherBY Private_Usresearcher
 /FORMAT=AVALUE TABLES
 /STATISTICS=CHISQ
 /CELLS=COUNT
 /COUNT ROUND CELL
 /BARCHART.
```

Crosstabs

Notes

Output Created		02-MAY-2016 12:53:44
Comments		
Input	Data	C:\Users\hpeoples\Box Sync\McGuire\Rothstein_Privacy\Results & Data Analysis\Analysis\ELSI Survey Complete SPSS Data Set.sav
	Active Dataset	DataSet1
	Filter	<none>
	Weight	<none>
	Split File	<none>
	N of Rows in Working Data File	1319
Missing Value Handling	Definition of Missing	User-defined missing values are treated as missing.
	Cases Used	Statistics for each table are based on all the cases with valid data in the specified range(s) for all variables in each table.
Syntax		CROSSTABS<br>/TABLES=Private_Outresearcher BY Private_Usresearcher<br>/FORMAT=AVALUE TABLES<br>/STATISTICS=CHISQ<br>/CELLS=COUNT<br>/COUNT ROUND CELL<br>/BARCHART.
Resources	Processor Time	00:00:00.13
	Elapsed Time	00:00:00.13
	Dimensions Requested	2
	Cells Available	349496

Case Processing Summary

	Cases					
	Valid		Missing		Total	
	N	Percent	N	Percent	N	Percent
How much do you / would you trust the following entities to keep your health information private? Academic researchers outside of the United States * How much do you / would you trust the following entities to keep your health information private? Academic researchers in the United States	1319	100.0%	0	0.0%	1319	100.0%

How much do you / would you trust the following entities to keep your health information private? Academic researchers outside of the United States * How much do you / would you trust the following entities to keep your health information private? Academic researchers in the United States Crosstabulation

Count

		How much do you / would you trust the following entities to keep your health information private? Academic researchers in the United States				Total
		Do Not Trust At All1	2	3	Trust Completely4	
How much do you / would you trust the following entities to keep your health information private? Academic researchers outside of the United States	Do Not Trust At All1	324	204	93	21	642
	2	3	197	152	21	373
	3	1	19	168	42	230
	Trust Completely4	0	1	7	66	74
Total		328	421	420	150	1319

Chi-Square Tests

	Value	df	Asymptotic Significance (2-sided)
Pearson Chi-Square	1098.472[a]	9	.000
Likelihood Ratio	989.120	9	.000
Linear-by-Linear Association	608.795	1	.000
N of Valid Cases	1319		

a. 0 cells (0.0%) have expected count less than 5. The minimum expected count is 8.42.

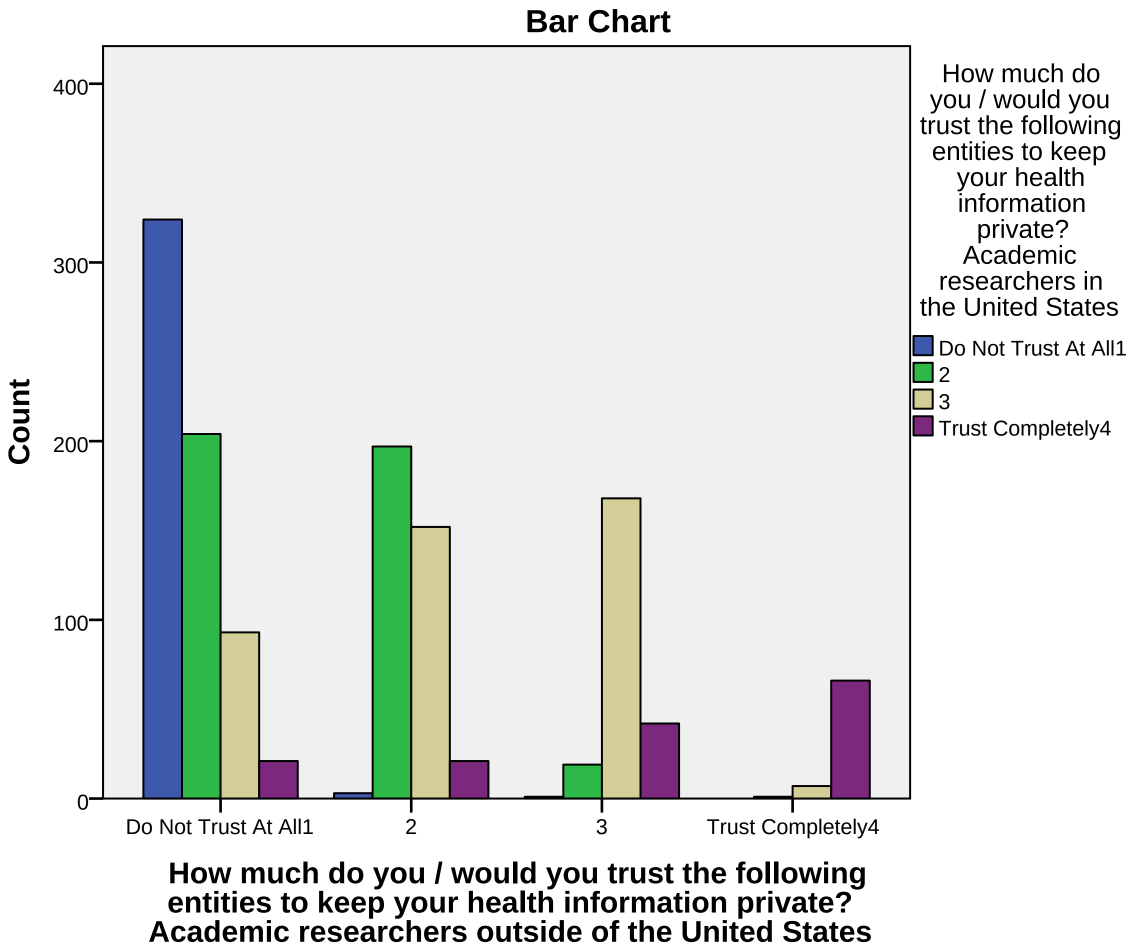


```
CROSSTABS
 /TABLES=Security_OutresearcherBY Security_Usresearcher
 /FORMAT=AVALUE TABLES
 /STATISTICS=CHISQ
 /CELLS=COUNT
 /COUNT ROUND CELL
 /BARCHART.
```

Crosstabs

Notes

Output Created		02-MAY-2016 12:54:04
Comments		
Input	Data	C:\Users\hpeoples\Box Sync\McGuire\Rothstein_Privacy\Results & Data Analysis\Analysis\ELSI Survey Complete SPSS Data Set.sav
	Active Dataset	DataSet1
	Filter	<none>
	Weight	<none>
	Split File	<none>
	N of Rows in Working Data File	1319
Missing Value Handling	Definition of Missing	User-defined missing values are treated as missing.
	Cases Used	Statistics for each table are based on all the cases with valid data in the specified range(s) for all variables in each table.
Syntax		CROSSTABS<br>/TABLES=Security_Outresearcher BY Security_Usresearcher<br>/FORMAT=AVALUE TABLES<br>/STATISTICS=CHISQ<br>/CELLS=COUNT<br>/COUNT ROUND CELL<br>/BARCHART.
Resources	Processor Time	00:00:00.16
	Elapsed Time	00:00:00.12
	Dimensions Requested	2
	Cells Available	349496

Case Processing Summary

	Cases					
	Valid		Missing		Total	
	N	Percent	N	Percent	N	Percent
How much do you / would you trust the following entities to keep your health information secure? Academic researchers outside of the United States * How much do you / would you trust the following entities to keep your health information secure? Academic researchers in the United States	1319	100.0%	0	0.0%	1319	100.0%

How much do you / would you trust the following entities to keep your health information secure? Academic researchers outside of the United States * How much do you / would you trust the following entities to keep your health information secure? Academic researchers in the United States Crosstabulation

Count

		How much do you / would you trust the following entities to keep your health information secure? Academic researchers in the United States				Total
		Do Not Trust At All1	2	3	Trust Completely4	
How much do you / would you trust the following entities to keep your health information secure? Academic researchers outside of the United States	Do Not Trust At All1	346	222	88	21	677
	2	2	219	135	19	375
	3	0	15	149	30	194
	Trust Completely4	0	1	5	67	73
Total		348	457	377	137	1319

Chi-Square Tests

	Value	df	Asymptotic Significance (2-sided)
Pearson Chi-Square	1197.791[a]	9	.000
Likelihood Ratio	1036.430	9	.000
Linear-by-Linear Association	619.419	1	.000
N of Valid Cases	1319		

a. 0 cells (0.0%) have expected count less than 5. The minimum expected count is 7.58.

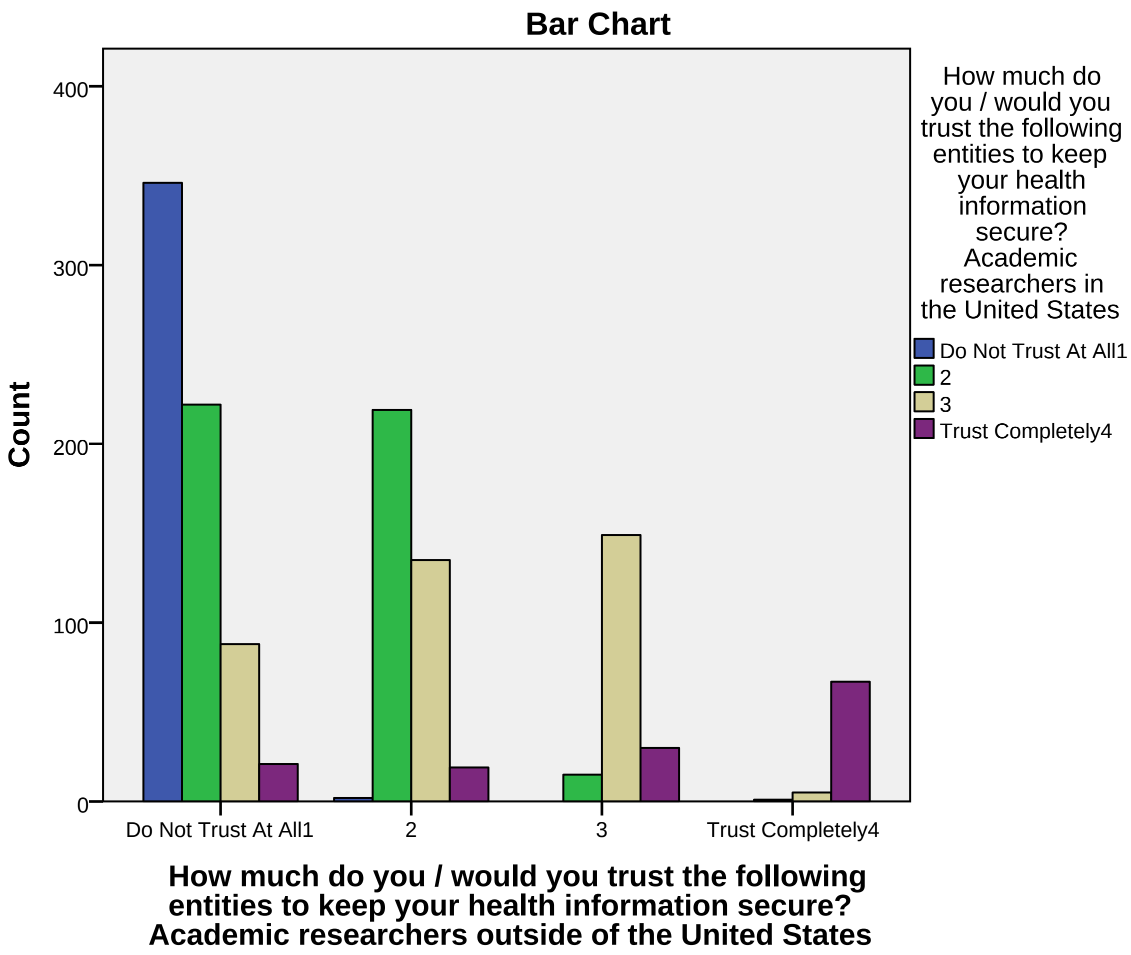
Bar Chart
Count
400
300
200
100
0
Do Not Trust At All1
2
3
Trust Completely4
How much do you / would you trust the following entities to keep your health information secure? Academic researchers in the United States
Do Not Trust At All1
2
3
Trust Completely4
How much do you / would you trust the following entities to keep your health information secure? Academic researchers outside of the United States
