# Supplementary material for: Beyond Our Borders? Public Resistance to Global Genomic Data Sharing
Source: PLoS Biol. 2016 Nov 2;14(11):e2000206. doi: 10.1371/journal.pbio.2000206 (PMC5091881; doi:10.1371/journal.pbio.2000206)
Supplement: S1 Text — (DOCX) [file pbio.2000206.s002.docx]

**Methods**

We developed a 45-item survey to assess participants’ social media usage, attitudes toward privacy and security of general online and health information, and generational differences in perspectives toward privacy and security of health information. All items used in the survey were novel measures except the Risk Propensity Scale (Meertens 2008) which was modified to a single item. Comfort levels and trust in United States (U.S.) and international academic researchers were also assessed and are reported in this manuscript. Novel measures were developed from a review of the literature and with expert input.

We conducted the survey online with the general public (n=1,319) in March 2016 in the U.S. using Amazon Mechanical Turk (MTurk). MTurk is an online marketplace that is well-known for crowdsourcing recruitment for survey research ([Buhrmester](http://mturk.com/mturk/welcome) 2011). Demographic characteristics of MTurk workers are fairly representative of the U.S. population, although they tend to be younger and better educated (Paolacci 2010). In order to control for data quality, we restricted participation to MTurk workers with high (≥92%) approval ratings, removed incomplete surveys from analysis, used attention check questions, including an instructional manipulation question, to avoid inattentive survey takers, and restricted participants from taking the survey more than once. (Peer 2014).

Descriptive statistics were generated for questions about participants’ comfort with their health information being accessed by academic researchers in and outside of the U.S.. Response options were on a 4-point scale consisting of Not at all comfortable, Not very comfortable, Somewhat comfortable, Very comfortable. Descriptive statistics were also calculated for questions about participants’ trust in academic researchers in and outside of the U.S. to keep their health information private and secure. Response options were on a 4-point scale anchored by Do not trust at all and Trust completely. All analyses were conducted using SPSS 24 (IBM Corp., New York, NY).

This study was approved by Baylor College of Medicine Institutional Review Board, Approval Number 4-37283. Informed consent was given when participants elected to take the survey after reviewing the following language:

We are inviting you to participate in a research study being conducted by Amy McGuire, JD, PhD and Mary Majumder, JD, PhD, in the Center for Medical Ethics and Health Policy at Baylor College of Medicine, and Mark Rothstein, JD in the Institute for Bioethics, Health Policy, and Law at the University of Louisville School of Medicine.

Participation in this study includes filling out an online survey about how you use social media, your views toward privacy and security of your information, and how you feel about different groups having access to your health information. We will also ask you some questions about yourself, including your age, race and ethnicity, education, and income. We will NOT ask you for any personally identifying information (like your name or email address). This survey should take you about 15 minutes to complete.

Your participation is completely voluntary, and you may stop at any time for any reason.

If you are willing to participate, please click the “Next” button below.

REFERENCES

Meertens et al. (2008). Measuring an Individual’s Tendency to take Risks: The Risk Propensity Scale. Journal of Applied Social Psychology 38(6) pp.1506-1520.

Buhrmester M, Kwang T, Gosling SD. Amazon’s Mechanical Turk: A new source of inexpensive, yet high-quality data? Perspect Psychol Sci. 2011; 6: 3-5.

Paolacci G, Chandler J, Ipeirotis PG. Running experiments on Amazon Mechanical Turk. Judgment and Decision Making 2010; 5(5): 411-419.

Peer E, Vosgerau J, Acquisti A. Reputation as a sufficient condition for data quality on Amazon Mechanical Turk. Behavior Research Methods 2014; 46(4): 1023-1031.
