# Supplementary material for: Beyond Our Borders? Public Resistance to Global Genomic Data Sharing
Source: PLoS Biol. 2016 Nov 2;14(11):e2000206. doi: 10.1371/journal.pbio.2000206 (PMC5091881; doi:10.1371/journal.pbio.2000206)
Supplement: S2 Text — (DOCX) [file pbio.2000206.s003.docx]

| **Table 1. Participant characteristics** | | |
| --- | --- | --- |
| **Characteristic - N (%) unless otherwise noted** | | **N=1319** |
| *Age* | | |
|  | Mean in years (SD) | 36.3 (12.4) |
| *Generational group^*^* | |  |
|  | Millennials (born after 1980, <35y.o.) | 772 (59) |
|  | Generation X (born 1965-1980, 51-36y.o.) | 348 (26) |
|  | Baby Boomers (born 1946-1964, 70-52y.o.) | 190 (14) |
|  | Silent Generation (born 1928-1945, 88-71y.o.) | 9 (0.7) |
| *Gender* | |  |
|  | Male | 661 (50) |
|  | Female | 651 (49) |
| *Race/Ethnicity* | |  |
|  | Hispanic or Latino | 101 (8) |
|  | Non-Hispanic white | 1025 (78) |
|  | African American | 73 (5) |
|  | Asian | 91 (7) |
|  | Non-Hispanic Other^†^ | 29 (2) |
| *Education* | |  |
|  | High school graduate/GED or less | 154 (12) |
|  | Some college or post-high school training | 450 (34) |
|  | College graduate or higher | 715 (54) |
| *Annual household income* | | |
|  | ≤ $49,000 | 719 (55) |
|  | $50,000 - $99,999 | 442 (34) |
|  | ≥ $100,000 | 158 (12) |
| *Health insurance source* | |  |
|  | Employer | 478 (36) |
|  | Parents or partner | 280 (21) |
|  | Healthcare Marketplace | 111 (8) |
|  | Medicaid or state insurance | 143 (11) |
|  | Medicare | 63 (5) |
|  | Private insurance | 61 (5) |
|  | Do not have health insurance | 157 (12) |
|  | Other | 26 (2) |
| *Political orientation* | | |
|  | Mean continuous scale, Liberal (-10) - Moderate (0) - Conservative (10) | -1.5 (4.2) |
| *Risk orientation* | |  |
|  | Mean continuous scale, Risk avoider (0) - Risk taker (10) | 4.1 (2.0) |
| ^*^Categories do not sum to 1319 because of participant non-response. | | |
| ^**^Generation groups defined by Pew Research Center : <http://www.pewresearch.org/methodology/demographic-research/definitions/> | | |
| ^†^Non-Hispanic Other includes: American Indian or Alaskan Native (13); Native Hawaiian or Other Pacific Islander (4), and Other (12) | | |
